# The real war on cancer: the evolutionary dynamics of cancer suppression

**DOI:** 10.1111/eva.12018

**Published:** 2012-10-10

**Authors:** Leonard Nunney

**Affiliations:** Department of Biology, University of CaliforniaRiverside, CA, USA and Center for Evolution and Cancer, University of CaliforniaSan Francisco, CA, USA

**Keywords:** adaptation, comparative biology, evolutionary medicine, evolutionary theory

## Abstract

Cancer is a disease of multicellular animals caused by unregulated cell division. The prevailing model of cancer (multistage carcinogenesis) is based on the view that cancer results after a series of (generally somatic) mutations that knock out the genetic mechanisms suppressing unregulated cell growth. The chance of these mutations occurring increases with size and longevity, leading to Peto's paradox: why don't large animals have a higher lifetime incidence of cancer than small animals? The solution to this paradox is evolution. From an evolutionary perspective, an increasing frequency of prereproductive cancer deaths results in natural selection for enhanced cancer suppression. The expected result is a prereproductive risk of cancer across species that is independent of life history. However, within species, we still expect cancer risk to increase with size and longevity. Here, I review the evolutionary model of cancer suppression and some recent empirical evidence supporting it. Data from humans and domestic dogs confirm the expected intraspecific association between size and cancer risk, while results from interspecific comparisons between rodents provide the best evidence to date of the predicted recruitment of additional cancer suppression mechanisms as species become larger or longer lived.

## Introduction

In 1971, the ‘war on cancer’ was initiated by Richard Nixon in the United States, but in reality, the war started with the evolution of multicellular animals more than half a billion years earlier. Cancer is a disease unique to multicellular animals and occurs when a tumour resulting from unregulated cell division invades other tissues. Cancer is not a problem for plants because the cell wall limits all forms of cell migration and hence prevents tumour cells from spreading (Michod 1996; Doonan and Sablowski 2010).

At first sight, cancer appears to be an evolutionary paradox: the somatic cells of a cancer are initially genetically identical to all the other cells of the individual, so the theory of kin selection (Hamilton 1964) predicts that the best evolutionary strategy is for all somatic cells to co-operate for the benefit of the germ line. However, there remains an inevitable conflict between the cellular level and the individual level that drives the occurrence of cancer (Michod 1996; Nunney 1999a). Conflict arises in this apparently benign scenario because of the general expectation that, although kin selection promotes cooperation over the long term when relatedness is high, cooperation is successful only if antisocial cheating can be prevented (Maynard Smith 1964). This result applies to the evolution of any organized social structure that depends upon the collaboration of many lower level units, each capable of independent replication. In the context of a multicellular animal, this conflict arises because a single cell can derive short-term success from its clonal proliferation within the individual, a success guaranteed given the abundance of resources available to a selfish cell surrounded by altruistic brethren. The result is cancer, a cellular strategy that is successful in the short term even though it ultimately dooms any chance of genetic transmission to future generations by killing the parent organism. For multicellularity to be successful, such antisocial acts had to be inhibited by suppression and/or policing (see Maynard Smith and Szathmáry 1995; Michod 1999).

Inhibiting cancer is a complex evolutionary problem because natural selection generally acts most effectively at the shortest timescale. For this reason, the antisocial somatic cells that create a cancer by maximizing their own short-term reproductive success are very difficult to stop. A population of cancer cells rapidly accumulates genetic variation and consequently substantial evolutionary potential to avoid the body's defences. At the same timescale, the body has no evolutionary options. Once a cancer has been initiated, pre-existing policing mechanisms (e.g. the immune system) are the only defence, while pre-existing suppression mechanisms (e.g. tumour suppressor genes) act earlier, reducing the chance of a cancer ever arising.

Developing these defences in advance requires that selection must operate at the longer timescale of the individual. This can be achieved through lineage selection (Nunney 1999a,b), a form of selection that promotes the stability of a social unit by favouring genetic mechanisms that suppress or police antisocial activity. In doing so, lineage selection can be effective in reversing the evolutionary advantage of short-term replicators. It requires that the social structures are discrete (i.e. no immigration of new unrelated replicators) and long-lived (relative to the generation time of the replicators). These conditions are satisfied for the case of an individual (the social structure) made up of cells (replicators), so we expect mechanisms of cancer suppression (preventing cancer cells arising) or policing (eliminating cancer cells once they arise) to be favoured whenever the occurrence of cancer significantly reduces the average individual fitness in the population.

But has not the evolutionary conflict between individuals and their cells been resolved? There has been plenty of time since the origin of multicellular animal for lineage selection to operate, and cancer is a relatively rare disease, except in individuals of postreproductive age, a pattern consistent with successful selection. However, this static view of cancer suppression overlooks a major problem that was articulated by Peto (1977) and named ‘Peto's paradox’ (Nunney 1999a). Peto (1977) observed that, because cancer is driven primarily by somatic mutation, large long-lived humans should have a much higher incidence of somatic mutations and hence of cancer than small short-lived mice, but they do not. Specifically, he noted that ‘a man has 1000 times as many cells as a mouse…. and we usually live at least 30 times as long as mice…. However, it seems that, in the wild, the probabilities of carcinoma induction in mice and in men are not vastly different. Are our stem cells really, then, 1 billion or 1 trillion times more “cancer proof” than murine stem cells? This is biologically pretty implausible; if human DNA is no more resistant to mutagenesis *in vitro* than mouse DNA, why don't we all die of notable carcinomas at an early age? Presumably some concomitant of our evolved ability to grow big and to live for three score years and ten is involved' (pp. 1413–1414).

Peto (1977) recognized that some change associated with evolving larger size and greater longevity had to be involved in keeping human cancer rates down, and presumed that it was a fortuitous correlated response of size and longevity changes. A number of such correlated responses have been proposed (reviewed by Caulin and Maley 2011); however, a major problem with these hypotheses is the lack of any explanation of why a fortuitous response to increased body size and/or longevity would alter the rate of cancer per cell in such a way that the overall incidence of cancer, scaled by lifespan, would stay relatively constant. For example, the (relative) age distribution of cancers in mice and humans is remarkably similar (Rangarajan and Weinberg 2003), despite their large difference in weight and longevity noted by Peto (1977).

A more plausible hypothesis is that whenever a cancer results in a significant loss of fitness in a population, then lineage selection will favour the spread of any variant that lowers the incidence of that cancer (Nunney 1999a). The result is a simple evolutionary solution to Peto's paradox that as specific tissues of a species become more susceptible to cancer due to increasing size or longevity, the resulting lowered fitness drives, via selection, the recruitment of additional cancer suppression mechanisms. Nunney (1999a) argued that such responses would generally be tissue-specific, directed at the cancer causing the greatest fitness loss. He developed a model to predict the relationship between the number of genes recruited to suppress cancer and the size and/or longevity of the source tissue.

Nunney (1999a) modelled cancer suppression based on the action of tumour suppressor genes and (proto)oncogenes; however, other mechanisms have been suggested as potential targets for resolving the paradox. Peto (1977) considered the possibility of DNA repair, but thought it unlikely to be important given the scale of the problem; however, modelling shows that a relatively small decrease in the somatic mutation rate can have a large effect if suppression is multigenic (Nunney 1999a). Cairns (1975) recognized the power of somatic mutation in driving cancer in a large rapidly dividing tissue such as the lining of the gut and proposed a number of potential changes that could evolve to reduce this effect, including having a limited number of localized stem cells with asymmetric division. Recently, DeGregori (2011) has revisited this idea and proposed that increased energy allocation in stem cells in longer lived and/or larger organisms may limit the accumulation of mutations, although no mechanism was specified. Other possibilities that have been proposed include telomere shortening (Nunney 2003), a possibility that now has strong support (Seluanov et al. 2007, 2008), as does increased cellular contact inhibition (Seluanov et al. 2009). These mechanisms and others were recently reviewed by Caulin and Maley (2011).

Cancer suppression involves two (sometimes overlapping) components. First, there are the genes directly involved in preventing unregulated cell division (the ‘gatekeepers’ of Kinzler and Vogelstein 1997). But these controls can be undermined by inherited and/or somatic mutations. This mutation-induced loss of regulation drives the first stages of cancer development: multistage carcinogenesis (see Weiss 2004). The frequency of inherited mutations is determined by a process of multigenic mutation-selection balance (Nunney 2003), a population-level phenomenon not controlled by individual genotypes; however, somatic mutation is controlled at the individual level. Minimizing somatic mutation requires a second group of genes (the ‘caretakers’; Kinzler and Vogelstein 1997) and involves error-free DNA replication, effective DNA repair, and the maintenance of appropriate epigenetic patterning (Sarkies and Sale 2012) and chromosomal structure (Stoler et al. 1999). Classified in either camp are some additional very important anticancer mechanisms such as the induction of apoptosis following DNA damage, a process typically involving what is arguably the most important cancer-controlling gene, *p53* (Levine and Oren 2009), and the erosion of telomeres due to the loss of telomerase activity (Garcia et al. 2007). If suppression fails, there is a final suite of cancer defences, the policing mechanisms, which are processes acting at a level higher than the single cell that can inhibit tumour progression. These include, most notably, the action of the immune system, as seen in the increased incidence of some cancers in immunosuppressed individuals (Boshoff and Weiss 2002), but also includes any role played by the healthy tissue around the tumour in limiting angiogenesis or other aspects of tumour growth.

## Predictions of the evolutionary model

The major predictions of the evolutionary model of cancer suppression developed by Nunney (1999a) are in essence very simple: an evolutionary increase in the size and/or longevity of a species will initially drive up the incidence of cancers; different cancers will increase in frequency to different degrees; and the cancers leading to a significant loss of fitness will drive selection to reduce their incidence via increased suppression. As a result, we expect some general patterns: that within any given species, the large rapidly dividing tissues will have more levels of cancer suppression than small slowly dividing ones; that between species, the large long-lived taxa will have more levels of suppression in a given tissue than small short-lived taxa; and that although the genes recruited to enhance suppression in different tissues and/or different species will be selected from a common pool of possible candidates, the specific controls recruited will depend upon the genetic variability available in the population at that particular moment in time. This last property of the model has the expected result that the spectrum of genes regulating different cancers in different tissues within a species may differ, and across species, tissue-specific suppression will share some similarities due to common ancestry but will diverge depending upon body size and longevity changes occurring in their lineages after divergence.

This basic framework leads to the second set of predictions related to population genetics of mutation-selection balance (Nunney 2003). A relatively subtle prediction is that very rare early-onset cancers will be primarily genetic (familial), while relatively common early-onset cancers will have a higher sporadic component. This prediction arises from the model because cancer suppression mechanisms are assumed to be recruited as discrete packages (e.g. an additional tumour suppressor gene). As a result, at any given time, some tissues may be relatively overregulated (very few sporadic cancers) and others under-regulated (a much higher frequency of sporadic cancers).

An important and robust prediction of the evolutionary model is that, although the overall prereproductive incidence of specific cancers is predicted to be independent of tissue size and turnover rate, postreproductive cancers will be most common in large rapidly dividing (typically epithelial) tissues (Nunney 2003). This last prediction is supported by the marked age-related shift in human cancers towards an increasing proportion of epithelial cancers in old age compared to younger adults (see DePinho 2000). Furthermore, this shift is expected to be more pronounced in larger animals, a prediction supported by the comparison of common age-related cancers in humans and mice. In humans, most are epithelial-origin carcinomas, while mice tend to develop mesenchymal origin lymphomas and sarcomas (Rangarajan and Weinberg 2004). Thus, although we cannot as yet compare the array of mechanisms that are involved in suppressing epithelial carcinomas to those suppressing lymphomas and sarcomas, this age-related shift seen in humans is consistent with the expectation that cancer suppression in large rapidly dividing epithelial tissues is the most complex.

Like any scientific model, the evolutionary model of cancer suppression needs rigorous testing. To help develop testable predictions, Nunney (1999a) modelled the accumulation of somatic mutations in the development of cancer during two stages of development, growth and stem cell maintenance, and here, we focus on the stage of postgrowth maintenance, because he noted that a simple approximation allows the growth phase to be incorporated into that model. The formula defining the probability of cancer when it is driven by the accumulation of *M* somatic mutations in a tissue of *C* cells during stem cell maintenance is:


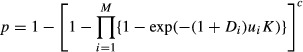
(1)

where *K* is the number of cell divisions, and for locus *i*, *u*_*i*_ is the somatic mutation rate and *D*_*i*_ = 0 if the locus is recessive (e.g. a classic tumour suppressor requiring two mutational ‘hits’ to remove its suppressive effect) or *D*_*i*_ = 1 if the locus is dominant (e.g. an oncogene). For small *p* (which is always the case in realistic scenarios), eqn (1) can be usefully (and very accurately) simplified to:


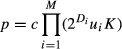
(2)

If we assume that all loci are recessive and have the same mutation rate, then eqn (1) reduces to:


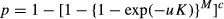
(3)

This special case of eqn (1) was independently derived by Calabrese and Shibata (2010), an identity that can be seen from their article by noting that, because *u* ≪ 1 and *k* > 1, then to a very close approximation (1−*u*)^*K*^ = exp(−*uK*). By analogy to eqn (2), this equation can be approximated by:


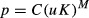
(4)

To fit these equations to human data on the age-specific incidence of cancer, we can use any of the eqns (1–4) to link age (*t*) and cancer rate (d*p*/d*t*), which historically we can consider equivalent to the death rate. Age can be explicitly included in these equations by replacing *K*, the number of divisions, by (*kt*), where *k* is the number of divisions/year, and *t* is age in years. Thus, using eqn (2), we have:


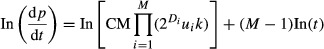
(5)

Simple multistage models are characterized by this linear relationship between ln(death rate) and ln(age) with a slope of *M*−1. For example, in the classic work of Armitage and Doll (1954), it was assumed that mutations had to occur in a specific order, but this does not alter the slope of the relationship. Nordling (1953) used death rate data for carcinomas in men to verify this linear relationship and obtained a slope of about 6. The more detailed cancer-specific analysis of Armitage and Doll (1954) provided additional support for a slope of 5–6 for some cancers (of the oesophagus, stomach, colon, rectum and pancreas), consistent with *M* = 6–7 mutations. Their estimates define the upper limits proposed for *M*.

At the lower extreme of *M*, Knudson (1971) proposed the two-hit model for retinoblastoma (i.e. control by the single tumour suppressor *Rb*). Retinoblastoma is a disease of early childhood that originates in the growing retina. The two-hit model (where *M* = 2) accurately accounts for the incidence of this disease (Hethcote and Knudson 1978), and Nunney (1999a) showed that the incidence of familial and sporadic forms could be accurately predicted from a variant of eqn (1) applicable to growing tissue. This equation shows that control by a single tumour suppressor gene provides adequate cancer suppression for tissues smaller than about 10^7^ cells (i.e. a few times larger than the tiny embryonic retina) provided it does not exhibit significant postgrowth division. The severe constraint on size and division strongly suggests that retinoblastoma is the only human cancer controlled by two-hit suppression (Nunney 2003).

The expected relationship between tissue size, somatic mutation rate and the level of suppression expected to evolve was shown in Nunney (1999a) for tissues where early-onset cancers arise during growth and for those tissues that divide continuously throughout life. A notable feature of these predictions is that the number of tumour suppressors (or equivalent) is not expected to be large, plausibly ranging from about 1 to 5 tumour suppressors (i.e. 2–10 mutational steps in a multistage model), although the occurrence of significant clonal expansion (see next paragraph) could raise the upper limit. In any event, these predictions are important because they suggest that cancer suppression is not a quantitative trait controlled by many independent loci of small effect. Thus, although it is not typically a simple trait controlled by just one gene, a change in the level of cancer suppression is expected to be a discrete change rather than part of a smooth continuum. This has important consequences for understanding the population genetics of cancer suppression (Nunney 2003).

The models of Armitage and Doll (1954) and Nunney (1999a) assume that cancer is a threshold event occurring after *M* mutations have accumulated in a cell. In general (but perhaps excluding retinoblastoma), it is probable that some clonal expansion (i.e. local tumour development) follows some or all of the mutational steps leading to cancer. Nunney (1999a) emphasized that such expansion can be approximated in the basic model (1) by increasing the mutation rates to match the effect of clonal expansion (e.g. increasing 1 cell to 100 cells increases the likelihood of all subsequent mutations by 100 fold). More explicit modelling of this process can take a variety of approaches. For example, Leubeck and Moolgavkar (2002) examined the incidence of colorectal cancer using a model in which clonal expansion only occurred late in the mutational progression but on a scale that substantially increased the probability of a final cancer-causing mutation, whereas Beerwinkel et al. (2007) assumed that each mutation in their multistage model resulted in a small increase in proliferative fitness. In any event, it is clear that precancerous proliferation of mutated cells can be an important factor in driving the later development of cancer; Brash (1997) emphasized this point with his suggestion that proliferation of surviving but mutated cells after the local death of skin cells following UV damage may be a factor in increasing the risk of skin cancer many years later.

## Human height and cancer

The evolutionary model of cancer suppression proposed by Nunney (1999a) predicts that natural selection will eliminate the association between the size of the species and cancer incidence. However, this adaptive process does not alter the size/cancer association within a species. Hence, one of the critical predictions of the model is an intra-specific relationship between increasing size and increasing cancer risk. Human size (as measured by height) varies considerably due to a combination of genetic and environmental factors. In mammals, such developmental differences typically reflect a difference in cell number (see Raff 1996; Lui and Baron 2011); hence, we expect tall individuals to have a higher cancer risk by virtue of their greater cell number, whereas weight added later in life as adipose tissue is generally related to increased cell size. Prior to the 1980s, most research related to human size focused on body weight, and results were somewhat contradictory; however, by the end of the decade, support for the possibility that height was a universal risk factor in human cancers had grown. At that time, Albanes and Winick (1988) proposed that within a species, the cancer risk depended on the number of cells and their rate of division, which are, of course, the properties modelled in eqns (1–4). This proposal was empirical: Albanes et al. (1988) found that height significantly increased overall cancer risk, and Albanes and Taylor (1990) presented evidence of a height effect in increasing the risk for a range of cancers. Specifically, they found significant effects for CNS, bladder and pancreatic cancers in both sexes, prostate, lung and colon cancers in men, and ovarian, uterine, rectal and breast cancers in women. Twenty years later, these initial conclusions are much more strongly supported as data from an increasing number of large studies have become available. In a meta-analysis of overall cancer risk using 11 large studies, Green et al. (2011) estimated a relative risk (RR) per 10 cm of height at 1.10 for men and 1.14 for women. They also examined the RR for specific cancers within the data from the Million Women study. Risk increased significantly for 10 of the 17 cancer categories identified, with only one showing a (nonsignificant) decrease with height (mouth/pharynx, 0.94).The three cancer categories with the highest RR were melanoma (1.32), kidney (1.29) and leukaemia (1.26).

There can be no doubt that tall humans are at a greater overall risk of cancer and that this risk is independent of the effects of obesity (as measured by BMI). For example, ovarian cancer risk increases with both height and BMI (Beral et al. 2012), while studies of receptor-positive breast cancer (John et al. 2010) and testicular cancer (Lerro et al. 2010) both showed a positive height relationship but a negative BMI effect.

Given such compelling evidence linking height and cancer risk in humans, we need to consider whether or not the effect observed is consistent with the general multistage model as represented by eqn (2). Specifically, we need to ask two questions. First, given the expected differences in cancer suppression between tissues discussed earlier, is the observation that the increased risk with height is very similar across very different types of cancer consistent with the model [as represented by eqn (2)]; and second, is the magnitude of the RR increase in overall cancer risk also consistent with the model? To examine these questions, we have to express RR in terms of the model: the RR of a 10 cm increase in height is *p*(cancer|height (*h* + 5)cm)/*p*(cancer|height (*h*−5) cm). It can be seen from eqn (2) that these two probabilities are identical except for the value of *C*, leading to the simple relationship:


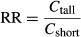
(6)

This relationship allows us to immediately answer the first question: the relative risk due to height is predicted to be independent of tissue type, at least in terms of the parameters critical to the likelihood of cancer: absolute size (*C*), rate of cell division (*K*), somatic mutation rate (*u*_*i*_) and the number (*M*) and nature (*D*_*i*_) of the genes involved in cancer suppression. However, some variation is expected because of varying allometric relationships among different organs, that is, variation across tissues in how cell number (*C*) scales with overall height. Regarding the second question concerning the magnitude of the effect, eqn (6) predicts that the RR values for general cancer risk associated with increased height directly reflect increases in cell number. On the basis of the Green et al. (2011) estimates, a 10-cm increase in height increases the overall risk of cancer by about 14% for women and 10% for men. A simple test of eqn (6) is whether or not these estimates lead to plausible values for human height. Assuming an exponent linking weight and height of 2 (the same exponent used in estimating the body mass index), these numbers suggest that a 10-cm increase in height represents roughly a 6.8% increase in height in women and a 4.9% increase in men, which predicts the average height of the two sexes to be 147 cm (4′ 10″) and 204cm (6′ 8′). These are not very satisfactory; however, recent estimates of the appropriate exponential scaling for women and men are 2.17 and 1.78, respectively, (Heymsfield et al. 2007) leading to much more reasonable heights of 161 cm (5′ 3″) and 182 (6′ 0″).

In summary, humans show a consistent increase in the risk of nearly all cancer types with increasing height, and the magnitude of this effect is consistent with what is predicted from a simple multistage model, that is, it is independent of tissue type and of a magnitude consistent with expectation.

## Body size and cancer risk in nonhumans

Artificial selection in domestic dogs has resulted in dramatic changes in body size. The high incidence of osteosarcoma in large dogs (see Withrow et al. 1991) has been cited as evidence supporting the basic tenet of the evolutionary model that larger size leads to a higher incidence of cancer (Leroi et al. 2003). The increase is dramatic, with more than a 150-fold increase in dogs weighing over 35 kg (Tjalma 1966). The increase may be directly related to the change in cell number; however, it can also be argued that osteosarcoma represents a special case linked to a pathology of the extended growth trajectory characteristic of large breeds (Withrow et al. 1991). For this reason, we need to examine whether or not there is an increase in the overall risk of cancer in large dogs. Addressing this question faces two important complications. First, it is possible that artificial selection for large size has already resulted in natural selection for increased cancer suppression. Dogs succumbing to early-onset cancer do not breed so that there is certainly the potential for additional cancer suppression to be favoured; however, on balance, we would expect that there has been too little time for natural selection to counteract the effects of larger size (Caulin and Maley 2011). Second, there is the complication of longevity. Large dog breeds are substantially shorter lived than small dog breeds (Michell 1999). This relationship is an ‘among breeds’ effect that does not hold within breeds (Galis et al. 2007) and hence appears to be a correlated response to size selection. As a result, comparing the lifetime cancer incidence of a breed close to its ancestral size (about 35 kg) to one that has been selected for a larger body size (e.g. 60 kg) is confounded by the difference in their lifespan. The decreased lifespan of large dogs is predicted to decrease the incidence of cancer and could easily mask a substantial increase in cancer risk due to their increased body size. The average sized breeds would typically have an expected life of around 11 years, while the larger ones live on average 2 years less (based on data from Greer et al. 2007). Using eqn (2) to define the RR for lifetime cancer of large dogs, we have:


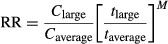
(7)

where *C* reflects size (weight) differences, and *t* reflects differences in lifespan. Substituting the size and lifespan for large and average dogs with *M* = 3 gives an RR of 0.94, that is, the lifetime cancer risk of the larger dog is less as a result of their shorter lifespan. This problem could be avoided by comparing cancer incidence up to a fixed age close to the maximum age for the large dogs, but substantially below the maximum for the smaller dogs, for example at 9 years; however, these data are not readily available.

**Figure 1:**
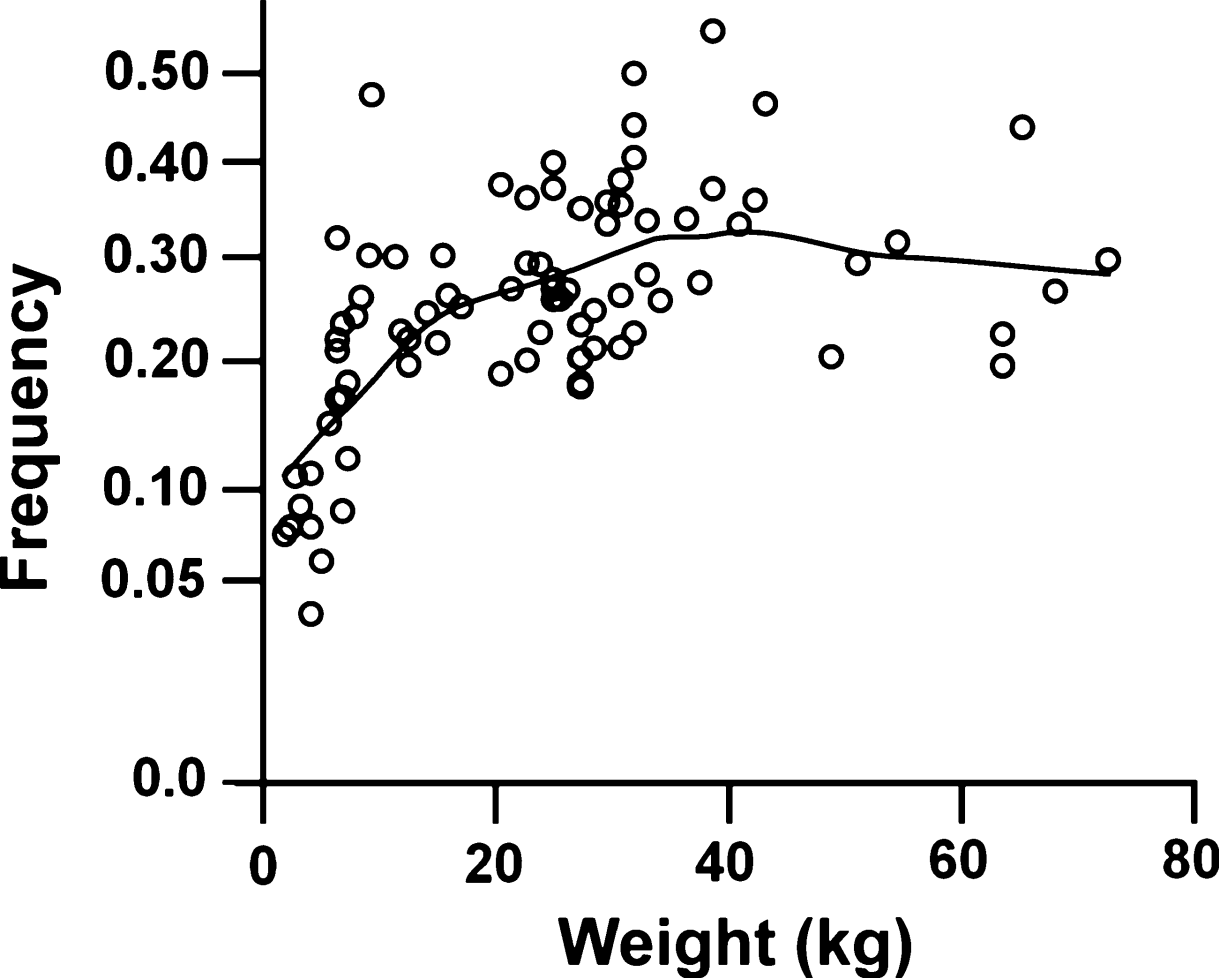
The frequency of death due to cancer in 82 breeds of domestic dog classified by their standard weight. Frequency is plotted on an arcsin (square root) scale that was used to fit a smoothed curve (Redrawn from Fleming et al. 2011).

The relationship between size and cancer susceptibility in large dogs is masked because their proportional size gain is small when compared to the reduction in their longevity raised to the power *M* (see eqn (7)). On the other hand, the proportional decrease in size seen in the smallest breeds of dog is substantial and can be expected to swamp the effect of their increased longevity. We can therefore predict that small dogs should exhibit a substantial decline in their lifetime incidence of cancer even though they have an extended lifespan. This reduction occurs because they are predicted to have more effective cancer suppression than is needed for their size and longevity, that is, they are overregulated. Under natural conditions, if a species is selected for smaller size, then this selection would be expected to result in the loss of excess regulation, either due to the fitness effects of the cost of overregulation or due to genetic drift resulting in some loss of function mutations becoming more common; however, these factors are unlikely to be of importance in small dog breeds given their short evolutionary history. Thus, a small dog of 5 kg, with a lifespan of about 13.5 years, has an RR (compared to the average sized dog) of 0.26 (for *M* = 3) and 0.40 (for *M* = 5). This prediction of a marked decline in cancer risk in small breeds is borne out in the analysis of Fleming et al. (2011). They analysed data linking the cause of death in a range of dog breeds to the breed weight. Their results show a sharp drop off in death due to cancer below 30–40 kg with a flat, slightly declining, relationship of cancer to weight above that range (Fig. 1).

The data from breeds of domestic dogs shown in Fig. 1, like the data from humans, are consistent with a critical prediction of the evolutionary model that an increase in size will increase cancer risk and hence result in selection for increased cancer suppression. The second critical prediction is that selection leading to a significant change in the size of species within a clade will result in predicable interspecific differences in cancer suppression.

One source of evidence on interspecific differences is in the comparison between human and mouse cells. It has long been known that mouse cells are sometimes easier to transform than human cells, and Rangarajan et al. (2004) demonstrated that, while just two pathways (involving p53 and Raf) need to be perturbed to immortalize mouse fibroblasts, an additional four pathways are involved (pRb, PP2A, telomerase and Ral-GEFs) for human fibroblasts.

However, the most compelling data on interspecific differences come from the comparative work of Seluanov et al. (2007, 2008, 2009) on rodents. Just as domestic dogs are ideal for studying intraspecific size variation, the rodent clade is ideal for studying interspecific variation in both size and longevity. Seluanov et al. (2007) examined one mechanism of cancer suppression, the repression of telomerase activity, in 15 species of rodent. Analysis of a range of somatic tissues showed that the largest rodents, capybara and beaver, showed a near complete repression of telomerase activity, while all small rodents showed high levels of telomerase activity. Given that the capybara and beaver are very distantly related, their telomerase suppression must have evolved independently.

The results of Seluanov et al. (2007) were consistent with an increase in body size leading to increased cancer suppression via telomerase repression; however, this mechanism showed no correlation with longevity. Seluanov et al. (2008) confirmed the telomerase results using cultured fibroblasts, demonstrating replicative senescence in the cells of the large rodents. They also showed that, in comparing short- and long-lived rodents that did not repress telomerase activity, cells growth rate was negatively correlated with longevity and uncorrelated with body size. They concluded that the mechanisms acting to suppress cancer in large versus long-lived rodents were different from the mechanisms acting in small versus large rodents. Further study of the very long-lived but small naked mole rat (with a maximum lifespan of > 28 years and body size of only about 35 gm) showed that their fibroblasts exhibit an unexpected second level of contact inhibition, a property that may be a powerful aid in cancer suppression (Seluanov et al. 2009). These results demonstrate precisely what is expected under the evolutionary model: the recruitment of additional but different mechanisms of cancer suppression in response to increased body size and increased lifespan.

## Discussion

An important medical debate concerns how much of our cancer is due to our environment, how much is due to our genotype and how much is just bad luck. Resolving this debate is important for understanding the occurrence of cancers in the young, for predicting the incidence of cancers in our increasingly ageing population, for developing efficient strategies for detecting the genes that inhibit our cancers and, ultimately, for preventing cancer. The evolutionary model, especially when combined with the theory of population genetics (Nunney 2003), can help us gain insight into what determines the frequency of different forms of cancer. A cancer is ‘nongenetic’ (sporadic) in origin when the set of cancer-causing mutations are due to somatic mutation alone. Environmental mutagens can drive this process; however, sometimes our baseline somatic mutation rate is sufficient. When this occurs, the cancer has no identifiable genetic or environmental cause and, as such, can be described as arising from bad luck. An evolutionary perspective clarifies the reasons why we should expect that sometimes (but only rarely) early-onset cancer will have no identifiable cause but that for some late-onset cancers, this lack of causation can be the rule.

A belief is sometimes expressed that the existence of cancer indicates that it must be in some sense beneficial; otherwise, natural selection would surely have eliminated it (e.g. Lichtenstein 2005; Garcia-Garcia 2009). In actual fact, even at the most superficial level, the incidence of cancer is broadly consistent with the action of natural selection acting against a detrimental pathology: prereproductive cancer is rare, while the incidence of postreproductive cancer is much higher. This pattern is expected because late-onset cancers have little or no effect on individual fitness (notwithstanding such factors as residual help to independent offspring and their offspring), and hence, their frequency is much less subject to the action of natural selection. However, the evolutionary model makes much more precise predictions than this. In this article, I have focused on two such predictions, the expected intraspecific increase in cancer risk with size and longevity and the expected absence of these correlations in interspecific comparisons. Others are also important. For example, DePinho (2000) considered that an important goal of cancer biology was to explain the marked age-related increase in carcinomas in humans. The evolutionary model predicts exactly this pattern that late-onset cancers will be predominantly those arising from tissues requiring the most layers of suppression, that is, epithelial tissues (Nunney 2003). On the other hand, the occurrence of early-onset cancers is determined primarily by the frequency of mutant alleles that predispose an individual to a given cancer. The frequency of such alleles in a population is determined by mutation-selection balance, and another set of predictions can be derived from the mutation-selection balance expected under multistage carcinogenesis (Nunney 2003).

Several reviews over the last 10 years have emphasized the value of incorporating an evolutionary approach into the study of cancer (Leroi et al. 2003; Crespi and Summers 2005; Merlo et al. 2006; Greaves 2007; Caulin and Maley 2011). Progress in gaining acceptance of these ideas has been slow, but this is understandable given the paucity of comparative data. However, the increasing availability of genomic and expression data in nonmodel organisms will aid in testing evolutionary ideas. We need to know how other animals, and especially very large animals (and of course whales are always mentioned in this context), suppress cancer. However, the work of Seluanov et al. (2009) on the naked mole rat illustrates how intriguing insights into potential methods of cancer prevention can be found in well-designed comparative studies of less spectacular groups of organisms.
